# The Four-Item Patient Health Questionnaire for Anxiety and Depression: A Validation Study in Infertile Patients

**DOI:** 10.22074/ijfs.2020.44412

**Published:** 2020-10-12

**Authors:** Azadeh Ghaheri, Reza Omani-Samani, Mahdi Sepidarkish, Mostafa Hosseini, Saman Maroufizadeh

**Affiliations:** 1 Department of Diabetes, Obesity and Metabolism, Reproductive Biomedicine Research Center, Royan Institute for Reproductive Biomedicine, ACECR, Tehran, Iran; 2 Department of Medical Ethics and Law, Reproductive Biomedicine Research Center, Royan Institute for Reproductive Biomedicine, ACECR, Tehran, Iran; 3 Department of Biostatistics and Epidemiology, School of Public Health, Babol University of Medical Sciences, Babol, Iran; 4 Department of Epidemiology and Biostatistics, School of Public Health, Tehran University of Medical Sciences, Tehran, Iran; 5 Department of Biostatistics, School of Nursing and Midwifery, Guilan University of Medical Sciences, Rasht, Iran

**Keywords:** Anxiety, Depression, Infertility, Reliability, Validity

## Abstract

**Background:**

The most common mental disorders in infertile patients are depression and anxiety. The four-item Patient Health Questionnaire-4 (PHQ-4) is a widely used tool that consists of the PHQ-2 depression and Generalized Anxiety Disorder-2 (GAD-2) scales. Given that PHQ-4 has not been validated in infertile patients, this study aimed to examine its reliability and validity in this population.

**Materials and Methods:**

Participants in this cross-sectional study consisted of 539 infertile patients from a referral fertility centre in Tehran, Iran. The PHQ-4, Hospital Anxiety and Depression Scale (HADS), World Health Organi- sation-Five Well-Being Index (WHO-5), Penn State Worry Questionnaire (PSWQ) and demographic/infertility ques- tionnaires were administered to all participants. Factor structure and internal consistency of PHQ-4 were evaluated via confirmatory factor analysis (CFA) and Cronbach’s alpha, respectively. The convergent validity of this scale was examined by its relationship with HADS, WHO-5 and PSWQ.

**Results:**

CFA results provided support for a two-factor model of PHQ-4. Internal consistency of the PHQ-4 and its subscales both were elevated with Cronbach’s alpha coefficients of 0.767 (PHQ-4), 0.780 (PHQ-2) and 0.814 (GAD-2). Inter-item correlations were between 0.386 and 0.639, and corrected item-total correlations were between 0.576 and 0.687. PHQ-4, PHQ-2 and GAD-2 showed positive correlations with measures of HADS-anxiety, HADS-depression, and PSWQ and neg- ative correlations with WHO-5, which confirmed convergent validity. Among demographic/fertility variables, we observed that gender, infertility duration, and failure in previous treatment were correlated with PHQ-4 and its subscales scores.

**Conclusion:**

The PHQ-4 is a reliable and valid ultra-brief screening instrument for measuring both anxiety and depres- sive symptoms in infertile patients.

## Introduction

Anxiety and depression, which tend to co-occur, are two of the most prevalent mental disorders in both the general population and outpatient settings (1, 2). Anxiety and depression are almost twice as common among people who experience fertility problems compared with the general population (3). This could be explained by the fact that infertility is considered as one of the great stressors in these people’s lives, which could lead to serious psychological, social and cultural consequences (4- 6). Among these, depression and anxiety are two of the most prevalent psychiatric disorders that adversely affect quality of life, well-being, and marital relationship and satisfaction (7, 8).

The results of a cross-sectional study on 1128 infertile patients showed a prevalence rate of 49.6% for anxiety and 33.0% for depression in Iran (8). A meta-analysis study in Iran also reported that the overall prevalence rate of depression among infertile couples was 0.47 (95% CI: 0.40, 0.55) (9). In another study, the prevalence of generalized anxiety disorder (GAD) was reported to be 28.3% among 1146 infertile patients in a referral fertility centre in Tehran, Iran (10).

In this regard, screening infertile patients for anxiety and depression could help predict those at risk and provide an opportunity for early intervention in order to improve quality of life in these patients. A brief screening tool that is both reliable and valid seems necessary as the first step of therapy for these disorders, especially in busy settings like referral infertility centres (11, 12).

The nine-item measure Patient Health Questionnaire-9 (the PHQ-9) has demonstrated strong psychometric properties for depressive disorders and has been widely used in numerous investigations (13-16). Similarly, the seven-item measure GAD-7 has shown good psychometric properties in assessing anxiety disorders in both the general population and clinic-based settings (17-19). In order to enhance efficiency and reliability of screening, the four-item ultra-brief PHQ-4 was developed. PHQ-4 consists of a two-item measure (PHQ-2) for depression and a two-item measure for anxiety (GAD-2). PHQ-4 and its subscales have also been shown to be an excellent self-reported screening tool for both depression and anxiety symptoms (12, 20, 21).

Thus far, the PHQ-4 has not been validated in infertile patients. The aim of this study was to assess the validity and reliability of PHQ-4 in infertile patients.

## Materials and Methods

### Participants and study design

This was a cross-sectional study of 539 infertile patients who were undergoing fertility treatment at the Infertility Treatment Centre of Royan Institute, Tehran, Iran. The data were collected in the evaluation phase of treatment by convenience sampling between May and August 2017. Those who were married were asked to complete the instruments separately from each other and refrain from discussing their answers. We followed the STROBE statement guidelines, whenever applicable, for reporting this study. The sample size was determined using the rule of thumb suggested by Comrey and Lee (22). They suggested that researchers obtain samples of 500 or more subjects whenever possible for factor analysis studies. The eligibility criteria were as follows: 1. suffering from infertility; 2. at least 18 years of age; 3. married and in a heterosexual relationship; and 4. able to read and write in Farsi. Further details of the design and methodology of this study have been described elsewhere (18, 23). This study was conducted after receipt of approval by the Ethics Committee of Royan Institute, Tehran, Iran (Registration Number: IR.ACECR.ROYAN.REC.1395.187), and all participants gave written informed consent to take part in this questionnaire-based study.

### Instruments

Demographic/infertility variables of participants that included age (years), sex (male, female), educational level (primary, secondary, university), duration of infertility (years), cause of infertility (self, partner, both/unexplained), failure of previous treatment (no, yes), and history of abortion (no, yes) were collected.

### Patient Health Questionnaire-4

The PHQ-4 is an ultra-brief tool for detecting both depression and anxiety disorders, which consists of the first two items of each of the measures PHQ-9 and GAD-7 (20). Hence, the PHQ-4 consists of two, 2-item subscales - one for depression (PHQ-2) and the Generalized Anxiety Disorder-2 (GAD-2) for anxiety. Each item is scored on a 4-point Likert scale that ranges from 0 (not at all) to 3 (nearly every day). The total PHQ-4 score ranges from 0 to 12, and total PHQ-2 and GAD-2 can range from 0 to 6. Higher scores denote greater levels of depression and anxiety. In this study, we used relevant translated items from PHQ-9 and GAD-7, which had been validated in infertile patients (18, 23).

### Hospital Anxiety and Depression Scale

The Hospital Anxiety and Depression Scale (HADS) is a commonly used self-administered tool that consists of 14 items. This scale is designed to measure both anxiety (HADS-A) and depression (HADS-D) (24). Each item is scored on a 4-point Likert scale that ranges from 0 to 3. The total HADS-A and HADS-D scores can range from 0 to 21, with higher scores denoting greater levels of anxiety and depression. The Persian version of HADS has demonstrated sound psychometric properties in infertile patients (25). In the present study, the Cronbach’s alpha coefficient of the HADS-A and HADS-D were 0.884 and 0.783, respectively

### World Health Organisation-Five Well-Being Index

The World Health Organisation-Five Well-Being Index (WHO-5) is a brief, 5-item self-administered tool that measures well-being during the previous two weeks (26). Each item is scored on a 6-point Likert scale that ranges from 0 (at no the time) to 5 (all of the time). The raw scores are transformed to a score from 0 to 100, with higher scores indicative of better well-being. The Persian version of the WHO-5 has demonstrated sound psychometric properties in infertile patients (27). In the present study, the Cronbach’s alpha coefficient of the WHO-5 was 0.858.

### Penn State Worry Questionnaire

The Penn State Worry Questionnaire (PSWQ) is a 16-item self-administered tool that measures both frequency and intensity of worry (28). Each item is scored on a 5-point Likert scale that ranges from 1 (not at all typical) to 5 (very typical). The total PSWQ score can range from 16 to 80, with higher scores denoting greater worry. We used the Persian-language version of PSWQ (with some modification in translation), which was validated among students (29). In the present study, the Cronbach’s alpha coefficient of the PSWQ was 0.886.

### Statistical analysis

The factor structure of the PHQ-4 was examined with confirmatory factor analysis (CFA) using maximum likelihood estimation. Two models were tested. The first model was a one-factor model with all four items loaded on single factor, which represented the PHQ-4 total score. The second model was a two-factor model where depression worded items were loaded on the PHQ-2 and the anxiety worded items were loaded on the GAD-2. Overall model fit was assessed using multiple fit criteria as suggested in the literature. Specifically, four goodness-of-fit indices were calculated - chi-square/degree of freedom (χ^2^/df), compara¬tive fit index (CFI), root mean square error of approximation (RMSEA), and standardised root mean square residual (SRMR). Values of χ^2^/df<2, CFI>0.95, and RMSEA and SRMR<0.08 indicate good fit to the data (30-33). The internal consistency of the PHQ-4 and subscale scores was evaluated by using Cronbach’s alpha, inter-item correlation and corrected-item total correlation. Convergent validity was examined by measuring the correlations between the PHQ-4 and measures of HADS-A, HADS-D, WHO-5 and PSWQ. Pearson’s correlation coefficient, independent t-test and one-way ANOVA were used to examine the relationship between PHQ-4 scores and demographic/fertility characteristics. Statistical analyses were conducted using IBM SPSS Statistics for Windows, Version 22.0 (IBM Corp., Armonk, NY, USA) and LISREL 8.80 (Scientific Software International, Inc., Lincolnwood, IL, USA). A P <0.05 was considered statistically significant.

## Results

### Participants´ characteristics

A total of 539 infertile patients (249 men and 290 women) participated in this study. The average age and infertility duration of the participants were 32.97 (SD=5.34) and 5.55 (SD=4.07) years, respectively. Table 1 summarizes the other demographic and fertility characteristics.

### Descriptive statistics and internal consistency of the Patient Health Questionnaire-4

Table 2 shows the item wording, descriptive statistics and internal consistency reliability of the PHQ-4 and its subscales. The item means ranged from 0.95 to 1.27. The mean (SD) scores were 4.63 (3.29) for the PHQ-4, 2.42 (1.86) for the PHQ-2 and 2.22 (1.82) for the GAD-2. The corrected item-total correlations for the PHQ-4 were in the acceptable range of 0.576 to 0.687. Moderate to strong inter-item correlations were observed among the PHQ-4 items (rs ranged from 0.386 to 0.639). Taking the brevity of the PHQ-4 and its subscales into account, we determined that the internal consistencies of the PHQ-4, PHQ-2 and GAD-2 were satisfactory (Cronbach’s Alpha=0.814, 0.767 and 0.780, respectively).

**Table 1:** Demographic and fertility characteristics of the participants


	Mean ± SD or n (%)

Age (Y)	32.97 ± 5.34
Sex	
Male	249 (46.2)
Female	290 (53.8)
Educational level	
Primary	92 (17.1)
Secondary	175 (32.5)
University	272 (50.4)
Duration of infertility (Y)	5.55 ± 4.07
Cause of infertility	
Self	163 (30.2)
Partner	155 (28.8)
Both/Unexplained	221 (41.0)
Failure of previous treatment	
No	253 (46.9)
Yes	286 (53.1)
History of abortion	
No	382 (70.9)
Yes	157 (29.1)


SD: Standard deviation (n=539).

**Table 2:** Item wording, descriptive statistics and internal consistency of the PHQ-4


	Mean (SD)	Corrected item total correlation	Alpha if item deleted	Cronbach’s Alpha

PHQ-2 items				
1. Little interest or pleasure in doing things	1.19 (1.05)	0.576	0.794	
2. Feeling down, depressed, or hopeless	1.22 (1.01)	0.687	0.741	
GAD-2 items				
3. Feeling nervous, anxious or on edge	1.27 (1.00)	0.674	0.748	
4. Not being able to stop or control worrying	0.95 (1.01)	0.600	0.782	
PHQ-2 total score	2.42 (1.86)			0.767
GAD-2 total score	2.22 (1.82)			0.780
PHQ-4 total score	4.63 (3.29)			0.814


SD; Standard deviation, PHQ-4; Patient Health Questionnaire-4, GAD-2; Generalized Anxiety Disorder-2, and PHQ-2; Patient Health Questionnaire-2.

### Convergent validity

As presented in Table 3, there were strong correlations between PHQ-4 and measures of HADS-A (r=0.717), HADS-D (r=0.535), WH0-5 (r=-0.559) and PSWQ (r=0.560). We obtained the same results for both the PHQ-2 and GAD-2. As seen in Table 3, the correlations of PHQ-2 with measures of depression (HADS-D and WHO-5) were higher than the correlations with measures of anxiety (HADS-A and PSWQ). The correlations of GAD-2 with measures of anxiety (HADS-A and PSWQ) were also higher than the correlations with measures of depression (HADS-D and WHO-5)

**Table 3:** Correlations between PQH-4 and measures of HADS, WHO-5, and PSWQ


	HADS-A	HADS-D	WHO-5	PSWQ

PHQ-2	0.573	0.491	-0.518	0.451
GAD-2	0.700	0.458	-0.475	0.545
PHQ-4	0.717	0.535	-0.559	0.560


PHQ-4; Patient Health Questionnaire-4, PHQ-2; Patient Health Questionnaire-2, GAD-2; Generalized Anxiety Disorder-2, HADS; Hospital Anxiety and Depression Scale, WHO-5; World Health Organisation-Five Well-Being Index, and PSWQ; Penn State Worry Questionnaire. All correlations were significant at the 0.001 level.

### Confirmatory factor analysis

The Confirmatory factor analysis (CFA) were used to examine the goodness of fit of the one and two-factor models of PHQ-4. The goodness of fit indices showed that the one-factor model did not fit the data well (χ^2^(2)=86.25, P<0.001; χ^2^/df=43.12; CFI=0.92; RMSEA=0.280 and SRMR=0.060). The result indicated that the two-factor model was a good fit to the data (χ^2^(1)=0.02, P=0.881; χ^2^/df=0.02; CFI=1.00; RMSEA<0.001 and SRMR=0.001). (Fig . 1)

**Fig.1:**
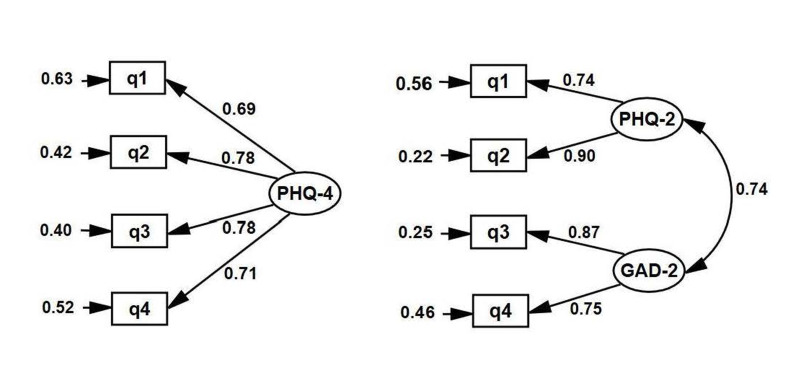
Confirmatory factor analysis (CFA) for a one- and two-factor model of the Patient Health Questionnaire-4 (PHQ-4).

### Relationship of the Patient Health Questionnaire-4 to demographic characteristics

Table 4 shows the relationships of the PHQ-4, PHQ-2 and GAD-2 with demographic/fertility characteristics. As seen in Table 4, women exhibited higher scores of PHQ-2, GAD-2 and PHQ-4 compared to men. Significant, but low, positive correlations were obtained between infertility duration and scores of PHQ-2 (r=0.118), GAD-2 (r=0.128) and PHQ-4 (r=0.139). Patients with previous treatment failures scored higher compared to patients who were undergoing their first treatment. Age, level of education, cause of infertility and history of abortion were not related to scores of the PHQ-4 and its subscales.

**Table 4:** Item wording, descriptive statistics and internal consistency of the PHQ-4


Variable	PHQ-2		GAD-2		PHQ-4	
	Mean (SD) or r	P value	Mean (SD) or r	P value	Mean (SD) or r	P value

Age (Y)	-0.014	0.741	-0.016	0.707	-0.017	0.691
Duration of infertility (Y)	0.118	0.006	0.128	0.003	0.139	0.001
Sex		<0.001		<0.001		<0.001
Male	1.90 (1.83)		1.78 (1.70)		3.67 (3.11)	
Female	2.86 (1.77)		2.59 (1.86)		5.45 (3.18)	
Educational level		0.731		0.063		0.218
Primary	2.54 (1.91)		2.62 (1.99)		5.16 (3.36)	
Secondary	2.35 (1.83)		2.10 (1.82)		4.46 (3.26)	
University	2.41 (1.86)		2.15 (1.76)		4.56 (3.24)	
Cause of infertility		0.300		0.934		0.554
Self	2.23 (1.90)		2.17 (1.95)		4.40 (3.36)	
Partner	2.49 (1.73)		2.23 (1.83)		4.72 (3.20)	
Both/unknown	2.50 (1.91)		2.24 (1.73)		4.74 (3.25)	
Failure of previous treatment		0.022		0.031		0.012
No	2.22 (1.88)		2.04 (1.79)		4.26 (3.24)	
Yes	2.59 (1.83)		2.37 (1.84)		4.96 (3.26)	
History of abortion		0.157		0.430		0.213
No	2.34 (1.81)		2.18 (1.82)		4.52 (3.21)	
Yes	2.59 (1.95)		2.31 (1.84)		4.90 (3.41)	


PHQ-4; Patient Health Questionnaire-4, PHQ-2; Patient Health Questionnaire-2, GAD-2; Generalized Anxiety Disorder-2, SD; Standard Deviation, and r; Pearson correlation coefficient.

## Discussion

To the best of our knowledge, this is the first study that examined the reliability and validity of the PHQ-4 in infertile patients. There is some evidence that infertile patients experience more anxiety and depression than the general population. In this study, the mean PHQ- 4 score was 4.63 (SD=3.29), which was considerably higher than reported in the German (M=1.76, SD=2.06) and Colombian (M=1.27, SD=2.01) general population (12, 34), US college students (M=2.98, SD=2.41) (21), patients from primary care clinics in the United States (M=2.5, SD=2.08) (20) and pre-operative surgical patients (M=2.63, SD=2.58) (35).

Taking the brevity of the PHQ-4 and its subscales into account, the internal consistency reliability of the PHQ-4 was relatively high. The obtained Cronbach’s alpha values were in line with previously reported values in different populations (12, 20, 21, 35). In addition, the inter-item correlations and corrected item-total correlations were also within acceptable ranges.

Despite the strong correlation between the PHQ-2 and GAD-2 subscales, CFA results demonstrated that these two subscales of the PHQ-4 reflected two separate dimensions (i.e., depression and anxiety). Previous exploratory factor analysis and CFA of the PHQ-4 also yielded two subscales, anxiety and depression (12, 20, 21).

Convergent validity of the PHQ-4 and its subscales was confirmed via its strong correlations with HADS, WHO- 5, and PSWQ inventories. In addition, the correlations of PHQ-2 (or GAD-2) with other depression (or anxiety) inventories were higher than the correlations with other anxiety (depression) inventories. These results were compatible with previous studies that reported correlations between PHQ-4 scores and measures of depression, anxiety, quality of life, well-being, hope and self-esteem (12, 20, 21, 34, 35).

We also examined the relationship between demographic/ fertility characteristics and the PHQ-4, PHQ-2 and GAD- 2. As expected, women exhibited higher scores of PHQ-2, GAD-2 and PHQ-4 compared to men. Empirical evidence supports the view that women express more anxiety and depression than men. Epidemiologic studies in the infertility context also show that anxiety and depression disorders are more prevalent among women than men (25, 36). Contrary to some general population-based studies (12, 34), there were no relationships between age and scores of PHQ-4 and its subscales. However, there were low indirect correlations between infertility duration and anxiety/depression scores. These results were consistent with previous studies (25, 37-39). In addition, there was a similar trend in other studies for measures of well-being, marital satisfaction and quality (25, 39). In our study, patients with unsuccessful treatment outcome obtained higher scores of PHQ-4 compared to patients who underwent their first treatment, which was in line with previous studies on measures of anxiety/depression and related measures such as quality of life, well-being and life satisfaction (39).

Several limitations of the current study should be mentioned. First, this was a single-centre study and the generalization of the findings may be limited. Second, unfortunately, structured diagnostic interviews based on DSM-IV were not performed, which precluded any discussion of the sensitivity and specificity of the scale. Third, because of the cross-sectional setting of the present study, causal inference between PHQ-4 scores and demographic/fertility characteristics could not be determined. Fourth, the test-retest reliability of the PHQ- 4 was not assessed in this study. Fifth, we did not have data on infertility-specific instruments such as fertility problem inventory (FPI) (40) and fertility quality of life (FertiQoL) (39) to examine convergent validity of the PHQ-4.

Despite the limitations, the present study provided a number of important implications for both researchers and practitioners. We assessed a sample of patients with infertility; therefore, our assessment of PHQ-4 suggests that this instrument can be used as a quick, reliable and valid primary screening instrument for patients who require in-depth assessment, follow-up for diagnosis and psychological intervention for anxiety and depression symptoms. Health professionals can use this scale to assess large numbers of infertile patients and rapidly screen them for anxiety and/or depression symptoms. Second, this questionnaire also provides a useful assessment tool when data must be collected by telephone or online. Third, clinicians and therapists who work with infertile patients should be aware of the factors associated with anxiety and/or depression symptoms such as female sex, long infertility duration and unsuccessful treatment.

## Conclusion

PHQ-4 is a reliable and valid screening instrument that can be used to measure anxiety and depressive symptoms in infertile patients. The scale is an ultra-brief and easy to use tool that can be administered in a few minutes. PHQ- 4 provides an economic tool for research and practice. Furthermore, the CFA results provide support for the twofactor structure of the scale (PHQ-2 and GAD-2) and use of these factors as discrete variables.
